# Hypothermia and its role in patients with ST-segment-elevation myocardial infarction and cardiac arrest

**DOI:** 10.3389/fcvm.2022.1051978

**Published:** 2022-11-29

**Authors:** Karsten Keller, Ingo Sagoschen, Volker H. Schmitt, Thomas Münzel, Tommaso Gori, Lukas Hobohm

**Affiliations:** ^1^Department of Cardiology, Cardiology I, University Medical Center Mainz, Johannes Gutenberg-University Mainz, Mainz, Germany; ^2^Center for Thrombosis and Hemostasis (CTH), University Medical Center Mainz, Johannes Gutenberg-University Mainz, Mainz, Germany; ^3^Medical Clinic VII, Department of Sports Medicine, University Hospital Heidelberg, Heidelberg, Germany; ^4^German Center for Cardiovascular Research (DZHK), Partner Site Rhine Main, Mainz, Germany

**Keywords:** myocardial infarction, STEMI, hypothermia, PCI, cardiac arrest

## Abstract

**Background:**

Patients suffering cardiac arrest resulting from ST-segment-elevation myocardial infarction (STEMI) are at very high risk of death. In addition to reperfusion strategies, therapeutic hypothermia is recommended for cardiac arrest patients who remain unconscious after resuscitation. However, data analysis of the impact of therapeutic hypothermia on survival showed inconsistent results. We aimed to investigate the benefits of therapeutic hypothermia in STEMI patients after cardiopulmonary resuscitation (CPR).

**Methods:**

Patients with STEMI who received CPR were identified after nationwide German inpatient data (2005–2019) were screened. These patients were stratified for therapeutic hypothermia. The impact of hypothermia on mortality and adverse in-hospital outcomes was analyzed.

**Results:**

Overall, 133,070 inpatients with STEMI and CPR (53.3% aged ≥70 years; 34% females) were recorded in Germany between 2005 and 2019, of which 12.3% (16,386 patients) underwent therapeutic hypothermia. Females (23.8 vs. 35.4%, *p* < 0.001) and patients aged ≥70 years (34.9 vs. 55.9%, *p* < 0.001) were less frequently treated with therapeutic hypothermia. The in-hospital case fatality rate was lower for STEMI with CPR and subsequent therapeutic hypothermia than for treatment without therapeutic hypothermia (53.5 vs. 66.7%, *p* < 0.001). Therapeutic hypothermia was independently associated with a reduced in-hospital case fatality rate {OR 0.83 [95% confidence interval (CI) 0.80–0.86], *p* < 0.001}. In addition, therapeutic hypothermia was associated with an increased risk for stroke (OR 1.37 [95% CI 1.25–1.49], *p* < 0.001), pneumonia (OR 1.75 [95% CI 1.68–1.82], *p* < 0.001), and acute kidney injury (OR 2.21 [95% CI 2.07–2.35], *p* < 0.001).

**Conclusion:**

Therapeutic hypothermia is associated with a survival benefit for STEMI patients after cardiac arrest.

## Highlights

- STEMI patients with cardiac arrest are at very high risk for death.
- Therapeutic hypothermia is recommended in patients who remain unconscious after resuscitation driven by cardiac arrest.
- Therapeutic hypothermia was associated with a survival benefit in STEMI patients after cardiac arrest.
- Therapeutic hypothermia was accompanied by an increased risk for stroke and pneumonia.

## Introduction

Coronary artery disease and its acute manifestation, myocardial infarction (MI), are the leading causes of death worldwide (1, 2). Approximately 3.8 million men and 3.4 million women die of coronary heart disease each year (1). In Europe, coronary heart disease accounts for almost 1.8 million deaths (20% of all deaths) per year, with large variations between countries (2). For patients with acute MI, immediate and successful myocardial reperfusion is the most effective strategy for reducing MI size and substantially improving the clinical outcome (1).

Patients suffering cardiac arrest resulting from ST-segment-elevation MI (STEMI) are at very high risk of death (2, 3). Several studies have shown that mild therapeutic hypothermia (between 32 and 36°C) increases survival after cardiac arrest (1, 2, 4), and is recommended after successful cardiopulmonary resuscitation (CPR) for STEMI-related cardiac arrest (2, 3, 5–8). The European Resuscitation Council Guidelines for Resuscitation 2015 recommended therapeutic hypothermia for adults after out-of-hospital cardiac arrest and who have an initial shockable rhythm and remain unresponsive after return of spontaneous circulation (ROSC) (6). In addition, therapeutic hypothermia should be recommended for adults after in-hospital cardiac arrest and who have some degree of initial rhythm and remain unresponsive after ROSC (6). Similarly, European Resuscitation Council Guidelines for Resuscitation 2021 recommended targeted temperature management for adults after out-of-hospital or in-hospital cardiac arrest, and who have some degree of initial rhythm and remain unresponsive after ROSC (7). In addition, the 2017 European Society of Cardiology (ESC) Guidelines for the management of acute MI in patients presenting with STEMI recommended the use of targeted temperature management for patients who remain unconscious after resuscitation from STEMI-related cardiac arrest (2). All three guidelines recommended the maintenance of a temperature between 32 and 36°C for at least 24 h (2, 6, 7). The German-Austrian AWMF guidelines 2020 concurred with the European Resuscitation Council Guidelines for Resuscitation with regard to therapeutic hypothermia, but suggested the maintenance of a temperature between 32 and 34°C for at least 24h (8).

Although the primary aim of hypothermia after cardiac arrest is to improve neurological outcomes, experimental studies reported a 10% reduction in myocardial infarct size for every 1°C decrease in body temperature (1, 9). However, other studies failed to uncover any beneficial effects regarding infarct size and survival (10–12).

## Methods

### Data source

Statistical analyses were performed on our behalf by the Research Data Center (RDC) of the Federal Bureau of Statistics in Wiesbaden, Germany. The RDC provided aggregated statistical results on the basis of SPSS codes, which we had previously created and sent to the RDC (source: RDC of the Federal Statistical Office and the Statistical Offices of the Federal States, DRG Statistics 2005–2019, own calculations) (13–15).

For the actual analysis of the nationwide German inpatient sample, we aimed to investigate the impact of therapeutic hypothermia (OPS code 8-607) on the outcomes of hospitalized patients with STEMI (ICD-codes I21.0, I21.1, I21.2, and/or I21.3) and who received CPR (OPS-code 8-77).

### Study oversight, ethical statement, and support

No commercial support was provided for this study, and the preparation of this paper was not externally influenced. As the authors of this paper only had access to summarized results provided by the RDC, and not individual patient data, approval by an ethics committee and patients' informed consent were not required, in accordance with German law (13, 14).

### Coding of diagnoses, procedures, and definitions

During 2004, diagnoses- and procedures-related remuneration was introduced and anchored into German hospitals. Coding of data regarding patients' diagnoses, coexisting conditions, surgeries, diagnostics, procedures, and interventions, according to the German Diagnosis Related Groups (G-DRG) system, was required, and the transfer of these codes to the Institute for the Hospital Remuneration System is now mandatory for German hospitals to receive reimbursement (15, 16). Patients' diagnoses have to be coded according the International Statistical Classification of Diseases and Related Health Problems (10th revision with German modification, ICD-10-GM) (17, 18). Additionally, diagnostic, interventional, and surgical procedures have to be coded using special OPS-codes (Operationen- und Prozedurenschlüssel). Thus, with this present analysis of the nationwide German inpatient sample, we were able to identify all hospitalized and coded STEMI patients with CPR in Germany during the observational period from 2005 to 2019. The selected STEMI patients with CPR were stratified for additionally coded therapeutic hypothermia.

### Study outcomes and adverse in-hospital events

The primary study outcome was to determine in-hospital case fatality (comprising all deaths, of any cause, during hospitalization). Additionally, we analyzed the prevalence of other adverse in-hospital events, such as pulmonary embolism (ICD-code I26), deep venous thrombosis and/or thrombophlebitis of the legs (ICD-code I80), pneumonia (ICD-codes J12-J18), acute kidney injury (ICD-code N17), ischemic or haemorrhagic stroke (ICD-codes I61-I64), gastro-intestinal bleeding (ICD-codes K92.0-K92.2), and need for transfusion of erythrocytes (OPS-codes 8-800).

### Statistical analysis

To compare STEMI patients with CPR treated with or without therapeutic hypothermia in Germany between 2005 and 2019, we analyzed differences between these two groups using a Wilcoxon–Whitney *U*-test for continuous variables and a Fisher's exact or chi^2^ test for categorical variables.

As therapeutic hypothermia was not consistently used over the study period for the treatment of MI, we analyzed time trends regarding its use. Temporal trends of STEMI patients with CPR during the observational period from 2005 to 2019, and the proportion and total of STEMI patients with CPR treated with therapeutic hypothermia over time, and with increasing age, were estimated. Linear regressions were used to assess these trends over time and the results are shown as beta (β) with corresponding 95% confidence intervals (CI).

Univariate and multivariate logistic regression models were calculated to investigate associations between therapeutic hypothermia and adverse in-hospital events. The multivariate regression models were adjusted in two different ways: adjustment I, adjusted for age, sex, obesity, cancer, heart failure, chronic obstructive pulmonary disease, essential arterial hypertension, hyperlipidaemia, acute and chronic kidney disease, diabetes mellitus, and atrial fibrillation/flutter; and adjustment II, including all parameters of adjustment I and coronary artery reperfusion strategies [including percutaneous coronary intervention (PCI) and/or coronary artery bypass graft].

We selected this epidemiological approach for the aforementioned adjustments to test the widespread independence of the impact of therapeutic hypothermia treatment on adverse in-hospital events from these known predictors of case fatality rate during hospitalization, as well as from the impact of differences in age, sex, and invasive and surgical reperfusion treatments. The results are presented as odds ratios (OR) and 95% confidence intervals (CI).

All statistical analyses were carried out using SPSS (IBM Corp. Released 2011. IBM SPSS Statistics for Windows, Version 20.0. IBM Corp: Armonk, NY, USA). Only *p* < 0.05 (two-sided) was considered to be statistically significant.

## Results

Overall, 133,070 hospitalizations of STEMI patients with CPR (53.3% aged ≥70 years; 34% females) were recorded in Germany between 2005 and 2019, of whom 12.3% (16,386 patients) underwent mild therapeutic hypothermia (Table 1).

**Table 1:** Characteristics, medical history, presentation, and outcomes of 133,070 STEMI patients with cardiopulmonary resuscitation, stratified for therapeutic hypothermia treatment.

**Parameters**	**STEMI and cardiopulmonary resuscitation without therapeutic hypothermia (*n =* 116,684; 87.7%)**	**STEMI and cardiopulmonary resuscitation with therapeutic hypo-thermia (*n =* 16,386; 12.3%)**	***p*-value**
Age	72 (61–79)	63 (54–73)	**<0.001**
Age ≥70 years	65,262 (55.9%)	5,726 (34.9%)	**<0.001**
Female	41,289 (35.4%)	3,900 (23.8%)	**<0.001**
In-hospital stay (days)	3 (1–12)	11 (2–21)	**<0.001**
**Recurrent myocardial infarction (during the first 4 weeks after previous myocardial infarction)**			
Recurrent myocardial infarction	1,200 (1%)	155 (0.9%)	0.325
**Traditional cardiovascular risk factors**			
Obesity	8,545 (7.3%)	1,110 (6.8%)	**0.011**
Diabetes mellitus	30,408 (26.1%)	4,003 (24.4%)	**<0.001**
Essential arterial hypertension	42,738 (36.6%)	5,730 (35%)	**<0.001**
Hyperlipidaemia	24,911 (21.3%)	4,040 (24.7%)	**<0.001**
**Comorbidities**
Heart failure	47,379 (40.6%)	7,923 (48.4%)	**<0.001**
Peripheral artery disease	7,292 (6.2%)	781 (4.8%)	**<0.001**
Atrial fibrillation/flutter	22,552 (19.3%)	3,344 (20.4%)	**0.001**
Cancer	4,139 (3.5%)	332 (2%)	**<0.001**
Chronic obstructive pulmonary disease	7,305 (6.3%)	872 (5.3%)	**<0.001**
Acute and chronic kidney disease	35,332 (30.3%)	6,943 (42.4%)	**<0.001**
Charlson comorbidity Index	5.35 ± 2.48	5.10 ± 2.40	**<0.001**
**Reperfusion treatments**			
Cardiac catheterization	70,793 (60.7%)	14,285 (87.2%)	**<0.001**
Percutaneous coronary intervention (PCI)	64,244 (55.1%)	13,649 (83.3%)	**<0.001**
Bare Metal Stent	23,686 (20.3%)	3,785 (23.1%)	**<0.001**
Drug Eluting Stent	34,534 (29.6%)	9,371 (57.2%)	**<0.001**
Bioresorbable vascular scaffold	119 (0.1%)	62 (0.4%)	**<0.001**
Coronary artery bypass graft	5,532 (4.7%)	268 (1.6%)	**<0.001**
**Adverse events during hospitalization**			
In-hospital death	77,819 (66.7%)	8,763 (53.5%)	**<0.001**
Pulmonary embolism	1,494 (1.3%)	239 (1.5%)	0.060
Deep venous thrombosis and/or thrombophlebitis	777 (0.7%)	228 (1.4%)	**<0.001**
Pneumonia	18,788 (16.1%)	4,789 (29.2%)	**<0.001**
Acute kidney injury	18,796 (16.1%)	5,299 (32.3%)	**<0.001**
Stroke (ischaemic or haemorrhagic)	3,745 (3.2%)	697 (4.3%)	**<0.001**
Gastro-intestinal bleeding	2,628 (2.3%)	563 (3.4%)	**<0.001**
Transfusion of blood constituents	25,430 (21.8%)	5,369 (32.8%)	**<0.001**

*p* values < 0.05 (two-sided) were considered to be statistically significant.

**Figure 1:**
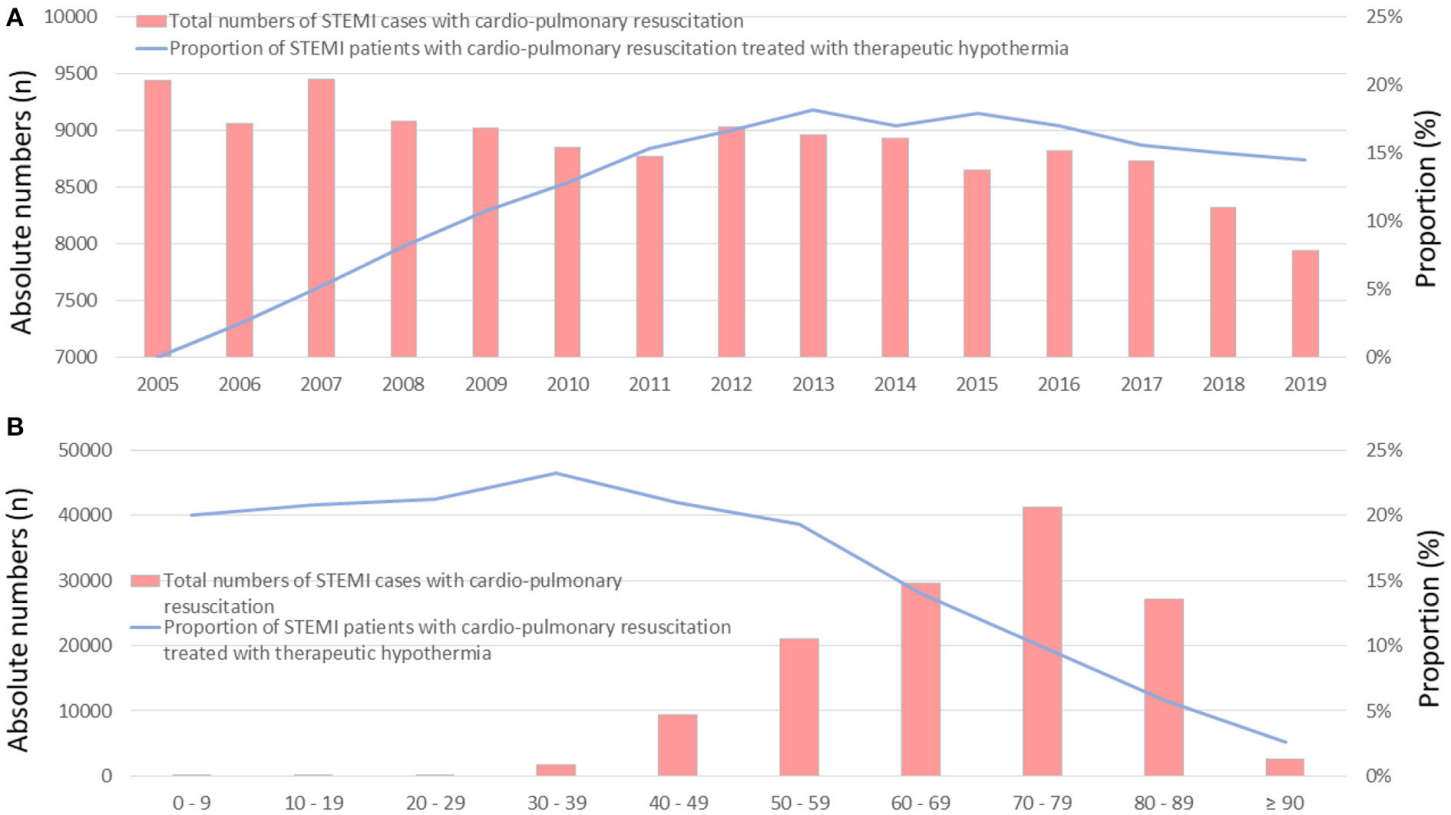
Time trends of total numbers of STEMI patients with CPR (red bars), and the proportion of these patients treated with therapeutic hypothermia (blue line) **(A)** time trends stratified by treatment years **(B)** time trends stratified by age/decade of life.

The total annual numbers of STEMI patients who underwent CPR and were treated with therapeutic hypothermia increased from 0 (0%) in 2005 to 7944 (14.5%) in 2019 [β 1.868 (95% CI 1.789 to 1.937), *p* < 0.001; Figure 1A]. The absolute numbers of patients who received hypothermia increased from the first to the sixth decade of life, and decreased afterwards, whereas the proportion of patients who underwent therapeutic hypothermia was essentially stable between the first and sixth decade, ranging from 19 to 25% (Figure 1B). There was an inverse association between increasing age and the utilization of therapeutic hypothermia [β −0.631 (95% CI −0.652 to −0.610), *p* < 0.001].

Female STEMI patients (23.8 vs. 35.4%, *p* < 0.001) and STEMI patients aged ≥70 years (34.9 vs. 55.9%, *p* < 0.001) were less frequently treated with therapeutic hypothermia than male and younger patients (Table 1). The median age of STEMI patients treated with hypothermia was 9 years younger [63.0 (54.0–73.0) vs. 72.0 (61.0–79.0) years, than that median age of STEMI patients not-treated with therapeutic hypothermia *p* < 0.001], and they were more likely to be male (76.2 vs. 64.6%, *p* < 0.001). Although heart failure was more prevalent in STEMI patients with CPR and therapeutic hypothermia (48.4 vs. 40.6%, *p* < 0.001), the Charlson Comorbidity Index was higher in patients without therapeutic hypothermia (5.35 ± 2.48 vs. 5.10 ± 2.40, *p* < 0.001), underlining a slightly aggravated comorbidity burden in those patients (Table 1).

STEMI patients with CPR and therapeutic hypothermia more frequently underwent invasive cardiac angiography (87.2 vs. 60.7%, *p* < 0.001) and were more often treated with PCI (83.3 vs. 55.1%, *p* < 0.001) in comparison to those without therapeutic hypothermia. In particular, drug eluting stents were used more often in STEMI patients with CPR and therapeutic hypothermia than in those without hypothermia (57.2 vs. 29.6%, *p* < 0.001; Table 1). By contrast, coronary artery bypass graft surgery was performed more often in resuscitated STEMI patients not treated with hypothermia (4.7 vs. 1.6%, *p* < 0.001).

The in-hospital case fatality rate was substantially lower in STEMI patients with CPR who were treated with therapeutic hypothermia than those not treated with hypothermia (53.5 vs. 66.7%, *p* < 0.001), despite a higher prevalence of adverse events, such as stroke (4.3 vs. 3.2%, *p* < 0.001), pneumonia (29.2 vs. 16.1%, *p* < 0.001), and acute kidney injury (32.3 vs. 16.1%, *p* < 0.001), in patients treated with hypothermia (Table 1). In addition, all investigated bleeding events were more prevalent in patients treated with therapeutic hypothermia.

The large survival difference between both groups might be the main reason for a longer length-of-hospital-stay in STEMI patients without vs. with therapeutic hypothermia [3.0 (1.0–12.0) vs. 11.0 (2.0–21.0) days, *p* < 0.001] (Table 1).

**Figure 2:**
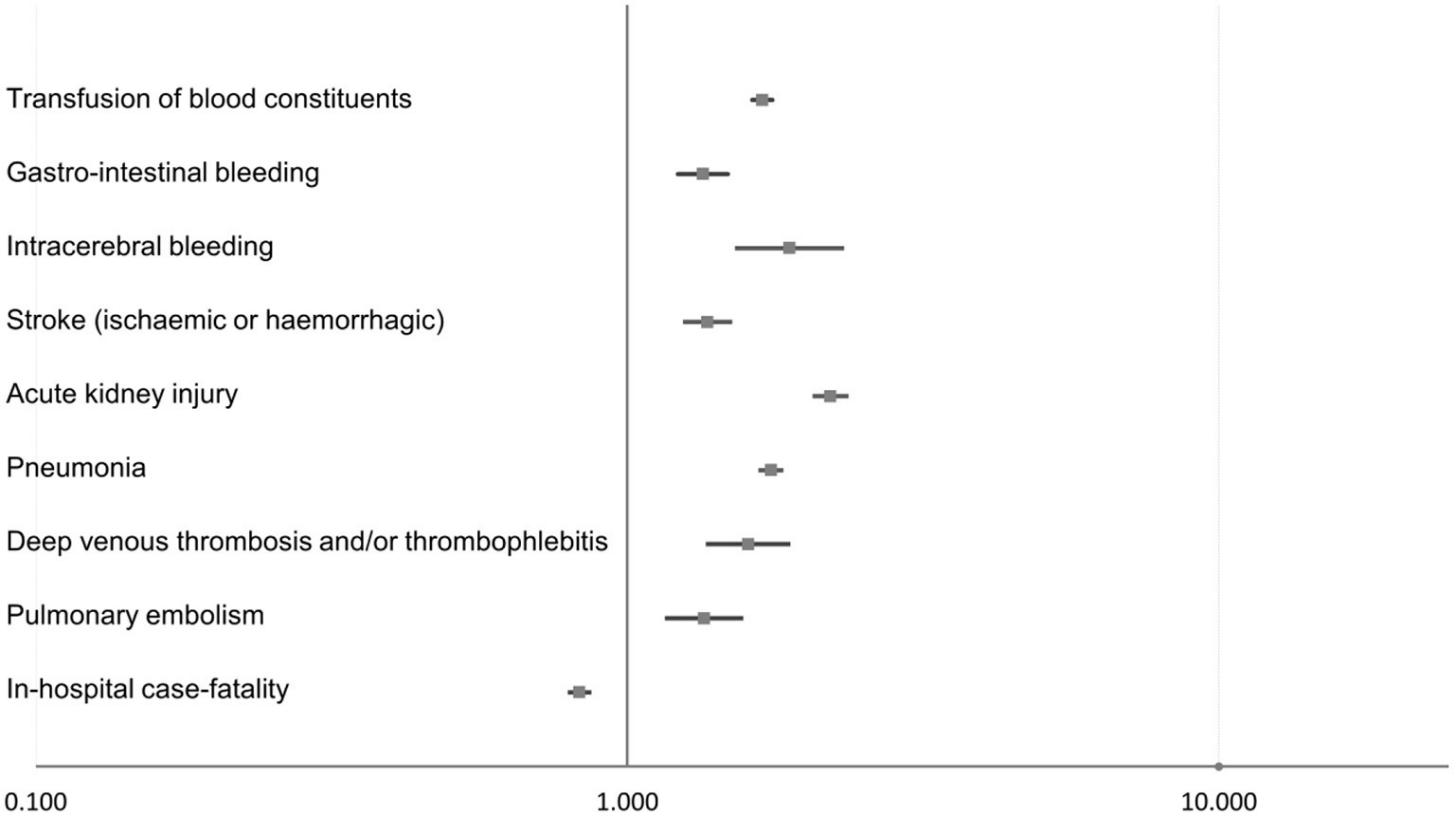
Impact of therapeutic hypothermia on adverse events during hospitalization in STEMI patients with CPR (multivariable logistic regression models).

Therapeutic hypothermia was independently associated with a reduced in-hospital mortality rate (OR 0.83 [95% CI 0.80–0.86], *p* < 0.001), as well as an increased risk of stroke (OR 1.37 [95% CI 1.25–1.49], *p* < 0.001), pneumonia (OR 1.75 [95% CI 1.68–1.82], *p* < 0.001), and acute kidney injury (OR 2.21 [95% CI 2.07–2.35], *p* < 0.001; Table 2, Figure 2).

**Table 2:** Impact of therapeutic hypothermia on adverse events during hospitalization in STEMI patients with cardiopulmonary resuscitation (univariable and multivariable logistic regression models).

	**Univariable regression model**		**Multivariable regression model (adjustment I)** ^*^		**Multivariable regression model (adjustment II)^†^**	
	**OR (95% CI)**	***p*-value**	**OR (95% CI)**	***p*-value**	**OR (95% CI)**	***p*-value**
In-hospital death	0.574 (0.555–0.593)	**<0.001**	0.736 (0.710–0.763)	**<0.001**	0.830 (0.800–0.862)	**<0.001**
Pulmonary embolism	1.141 (0.995–1.309)	0.060	1.120 (0.973–1.290)	0.116	1.348 (1.167–1.557)	**<0.001**
Deep venous thrombosis and/or thrombophlebitis	2.105 (1.814–2.442)	**<0.001**	1.660 (1.423–1.935)	**<0.001**	1.601 (1.369–1.872)	**<0.001**
Pneumonia	2.152 (2.073–2.233)	**<0.001**	1.840 (1.767–1.915)	**<0.001**	1.750 (1.680–1.822)	**<0.001**
Acute kidney injury	2.489 (2.400–2.581)	**<0.001**	2.287 (2.151–2.431)	**<0.001**	2.205 (2.073–2.347)	**<0.001**
Stroke (ischaemic or haemorrhagic)	1.340 (1.234–1.455)	**<0.001**	1.234 (1.133–1.343)	**<0.001**	1.367 (1.253–1.491)	**<0.001**
Gastro-intestinal bleeding	1.544 (1.408–1.694)	**<0.001**	1.360 (1.236–1.497)	**<0.001**	1.342 (1.218–1.479)	**<0.001**
Transfusion of blood constituents	1.749 (1.688–1.812)	**<0.001**	1.500 (1.443–1.558)	**<0.001**	1.691 (1.625–1.760)	**<0.001**

* Adjustment I: adjusted for age, sex, obesity, cancer, heart failure, chronic obstructive pulmonary disease, essential arterial hypertension, hyperlipidaemia, acute, and chronic kidney disease, diabetes mellitus, and atrial fibrillation/flutter.

† Adjustment II: Adjusted for age, sex, obesity, cancer, heart failure, chronic obstructive pulmonary disease, essential arterial hypertension, hyperlipidaemia, acute and chronic kidney disease, diabetes mellitus, atrial fibrillation/flutter, and coronary artery reperfusion strategies (including PCI and/or coronary artery bypass graft). *P* values < 0.05 (two-sided) were considered to be statistically significant.

When focusing on the subgroup of STEMI patients with CPR and coded mechanical ventilation, therapeutic hypothermia was also accompanied by reduced in-hospital case fatality (*p* < 0.001). The association between therapeutic hypothermia and decreased in-hospital case fatality was strong in this patient group and independent of the adjusted parameters (univariate regression, OR 0.403 [95% CI 0.342–0.476], *p* < 0.001; multivariate regression – adjustment I, OR 0.455 [95% CI 0.383–0.541], *p* < 0.001; multivariate regression – adjustment II, OR 0.530 [95% CI 0.443–0.633], *p* < 0.001).

## Discussion

The objective of the present study was to analyze the impact of therapeutic hypothermia on in-hospital case fatality in a very large nationwide sample of more than 130,000 STEMI patients who suffered cardiac arrest.

The results can be summarized as follows:

i) Only 12.3% of the 133,070 hospitalizations of STEMI patients with CPR underwent therapeutic hypothermia.
ii) The proportion of STEMI patients with CPR treated with therapeutic hypothermia increased substantially between 2005 and 2019.
iii) Therapeutic hypothermia was used more frequently with younger STEMI patients with CPR (first to sixth decades) than with MI patients aged 60 years and older.
iv) Therapeutic hypothermia positively affected the case fatality rate of STEMI patients with CPR (OR of 0.83 for reduced mortality), independent of age, sex, comorbidities, and reperfusion treatments.
v) The adverse in-hospital events stroke, pneumonia, and acute kidney injury were detected more often in STEMI patients who underwent CPR and were treated with therapeutic hypothermia.

Patients who are unconscious after cardiac arrest are at substantial risk of death and developing neurological deficits (2, 3, 16). The post-cardiac arrest syndrome comprises different organ dysfunctions, including brain injury, myocardial dysfunction, and systemic ischemia/reperfusion syndrome (17, 19, 20). Brain injury is one of the most critical aspects and the leading cause of death in the post-cardiac arrest period (18, 19, 21). Thus, neuroprotective strategies are crucial after successful out-of-hospital resuscitation of cardiac arrest patients. Besides therapeutic hypothermia and coronary revascularization, optimal intensive care unit management with regard to sedation and ventilation, blood glucose management, the prevention and adequate treatment of epileptic seizures, and pharmacological strategies is of utmost importance for improving outcomes in these critically ill patients (18, 19). Targeted temperature management is the main neuroprotective tool in post-cardiac arrest therapy (18, 19). Current and recent guidelines recommend a mild therapeutic hypothermia aiming a constant temperature between 32 and 36°C for at least 24 h in patients who remain unconscious after CPR from cardiac arrest of presumed cardiac cause (2, 5–8, 22). In particular, therapeutic hypothermia should be initiated in STEMI patients suffering out-of-hospital cardiac arrest, and therefore, cooling should be started before, or at least at the time of, cardiac catheterization (22).

Our results demonstrated a substantial survival benefit in STEMI patients who underwent CPR and were treated with therapeutic hypothermia compared with those who did not receive this therapy. Although successful early myocardial reperfusion is the most important and effective strategy for reducing MI size and substantially improving clinical outcomes, including MI patient mortality (1, 2, 12, 22–25), our study shows that therapeutic hypothermia reduced the case fatality rate in STEMI patients post cardiac arrest by 13.2% (from 66.7 to 53.5%) compared with those patients who did not receive the therapy. As therapeutic hypothermia was used more often in younger STEMI patients between the first and sixth decades of life, males and STEMI patients post cardiac arrest who did not receive therapeutic hypothermia had a slightly aggravated comorbidity profile and were less often treated with PCI. As such, we used multivariate regression models to adjust for these important differences between patient groups. Nevertheless, the multivariate regression model still showed a substantially reduced in-hospital case fatality rate (OR 0.83, 95% CI 0.80–0.86). However, it is important to note that the exact timing and course of CPR (i.e. whether CPR was performed on admission, early on during a patient's hospitalization before cardiac catheterization, or when a complication arose during their stay in hospital), as well as the timepoint and the duration of hypothermia treatment, could not be determined and was thus a limiting factor in potential analyses. To circumvent this problem, we additionally analyzed the effect of hypothermia in STEMI patients who underwent CPR and had to be mechanically ventilated. In this group, the positive effect of hypothermia on in-hospital case fatality was still consistently apparent.

Our results are in accordance with several studies, showing that therapeutic hypothermia is accompanied by better survival and neurological outcomes in patients with cardiac arrest (1, 2, 4, 26, 27). In several studies, therapeutic hypothermia was consistently accompanied by a reduction in infarct size (1, 9, 27). In addition, experimental studies with animals revealed that therapeutic hypothermia was dose-dependently cardioprotective during ischaemia but not after reperfusion (27). Nevertheless, the early clinical studies ICE-IT and COOL-MI were not able to identify a beneficial role for hypothermia, possibly due to the slow cooling approach used (26–29). By contrast, a combination of cold saline and induced endovascular cooling reduced infarct size 3 days after an MRI-measured MI index event in a pilot study of 20 STEMI patients (26).

One study of the ‘hypothermia after cardiac arrest study group’ reported that therapeutic mild hypothermia was accompanied by an increased rate of favorable neurologic outcomes and reduced mortality in patients successfully resuscitated (ventricular fibrillation) after cardiac cause-related cardiac arrest (4). The risk ratio in this study was 0.74 (95% CI 0.59–0.95) with regard to mortality within 6 months after cardiac arrest (4). Owing to the different follow-up observational periods, it is difficult to compare risk ratio with the OR of our study. However, as the early phase up to patients' discharge is the most critical in terms of outcome (21, 30), a partial comparison of the study results might be possible.

Despite these positive effects, therapeutic hypothermia treatment might be associated with slow uptake, a delayed onset of action and perhaps reperfusion, and a diminished oral antiplatelet agent effect (2). Bednar et al. (31) reported that prasugrel, as well as ticagrelor, but not clopidogrel, were effective at inhibiting platelets in MI patients treated with therapeutic hypothermia after cardiac arrest. Prasugrel and ticagrelor were highly effective, whereas clopidogrel was not effective at inhibiting platelets, even at an increased dose (31). In line with these findings, ticagrelor provided significantly faster and stronger platelet inhibition than clopidogrel in comatose survivors of out-of-hospital cardiac arrest undergoing PCI and hypothermia (32). Crushed and dissolved ticagrelor, and prasugrel tablets, injected by naso-gastric tubes, are therefore simple and effective options for achieving fast and persistent P2Y12 inhibition (32–34). By contrast, other results from the ISAR-SHOCK registry suggested that therapeutic hypothermia does not negatively impact platelet reactivity in shock patients receiving either clopidogrel or prasugrel (35, 36). However, the notion that cooling should not delay primary PCI is uncontroversial and of great interest, as is anticoagulation (2).

Other studies failed to reveal beneficial effects regarding infarct size and survival (3, 10–12). A study by Testori et al. (12) showed that out-of-hospital induced therapeutic hypothermia as an adjunct therapy to primary PCI did not improve myocardial salvage in STEMI patients (12). In parallel, Dixon et al. (10) reported that endovascular cooling can be performed safely as an adjunct to primary PCI for acute MI, and although median infarct size was non-significantly smaller in patients who received cooling, there was no change in major adverse cardiac and cerebrovascular events (10). Additionally, Chan et al. (11) could not identify a survival benefit of therapeutic hypothermia compared with usual care (11). Of 26,183 patients successfully resuscitated, 1,524 patients treated with therapeutic hypothermia were propensity score matched to 3,714 patients who did not receive hypothermia treatment. By contrast to the these studies, therapeutic hypothermia was even associated with a lower likelihood of in-hospital survival, and had a lower likelihood of favorable neurological survival (11). In addition, Nielsen et al. (3) demonstrated that therapeutic hypothermia at a targeted temperature of 33°C does not confer an additional benefit for unconscious survivors of out-of-hospital cardiac cause-related cardiac arrest compared with a targeted temperature of 36°C (3). Nevertheless, some specific characteristics of this study have to be mentioned: first, the average time to reach the target temperature was more than 9 h after ROSC; (3) second, cooling was not consistently achieved using surface cooling alone, but also by using intravenous fluids; (3) and third, patients underwent neurological prognostication with new, untested, and non-validated prediction models, resulting in a high rate of early life support withdrawal, which might have contributed to equal mortality rates in both groups (3).

In spite of these inconsistent and, in part, controversial study results, large nationwide studies, such as the present study, are needed to provide clear evidence and support the formulation of treatment recommendations. The present study, which included more than 130,000 STEMI patients with CPR, highlights a substantial survival benefit from adding therapeutic hypothermia to revascularization strategies, and therefore, strongly supports ESC guideline recommendations (2). Nevertheless, we have to be aware of the aforementioned limitations regarding the exact timing and course of the underlying events/treatments during hospitalization.

Differences regarding length of hospital stay and frequency of PCI treatment between the groups that had a shorter hospital stay, as well as the lower frequency of PCI in STEMI patients post cardiac arrest who did not receive therapeutic hypothermia, suggest that STEMI patients not receiving therapeutic hypothermia die, in not-insignificant part, very soon after hospital admission, and therefore, cannot be treated by revascularization before their in-hospital death. In STEMI patients, therapeutic hypothermia might prolong the therapeutic window after CPR for the safe application of revascularization strategies before the recurrence of cardiac arrest, and thus is associated with benefits in outcomes. In addition, STEMI patients who were not unconscious after CPR, and therefore did not undergo hypothermia, also contributed to the low overall rate of hypothermia treatment.

Multiple organ dysfunction syndrome is common after cardiac arrest (20, 37). Following out-of-hospital cardiac arrest, the early post-resuscitation phase is accompanied by abnormalities in microcirculation and peripheral tissue perfusion, resulting from vasoconstriction, due to induced systemic hypothermia, and impaired systemic blood flow (17). Persistence of these alterations results in organ failure and death, independent of systemic hemodynamics (17). Notably, in severely injured trauma patients, the early post-resuscitation phase is a significant risk factor for multiple organ dysfunction syndrome but not mortality (38).

As expected, and in accordance with previously published studies, pneumonia occurred more often in STEMI patients treated with therapeutic hypothermia (39, 40). This occurrence might also be caused by prolonged sedation to achieve hypothermia in those intention-to-treat patients (39, 41). In this patient group, pneumonia was associated with prolonged respiratory support and intensive-care-unit stay, but did not significantly impact mortality (39). In addition, the higher prevalence of acute kidney injury in patients with mild therapeutic hypothermia might be attributed to the higher rate of cardiac catheterization. PCI and targeted temperature management are useful interventions for improving myocardial function, survival, and neurological outcomes, but have numerous cardiovascular and renal effects, and it is still unclear whether their net effect on renal function is beneficial or harmful (42, 43). Therapeutic hypothermia induces peripheral vasoconstriction accompanied by temporary relative central hypervolemia, with increased renal blood flow and diuresis mediated by the suppression of vasopressin (42). Subsequently, low circulating volume and high afterload might cause hypotension and reduced renal blood flow, with vasoconstriction of afferent arterioles potentially further decreasing renal blood flow and increasing renal vascular resistance (42). Consequently, renal function, which is already impaired due to imminent cardiac arrest, can become progressively depressed, resulting in acute kidney injury (42).

Although therapeutic hypothermia in conjunction with primary coronary revascularization is associated with favorable neurological outcomes in the large majority of STEMI patients surviving out-of-hospital cardiac arrest (44, 45), our study revealed a higher stroke rate in STEMI patients treated with therapeutic hypothermia. This might be a result of the lower case fatality rate during the initial phase (first 2 days) and the longer length of in-hospital stay of STEMI patients treated with therapeutic hypothermia than STEMI patients who did not receive treatment, which are sequelae of higher early mortality rates in STEMI patients who do not receive therapeutic hypothermia.

In accordance with previously published data, our study revealed a decrease in the total annual number of STEMI patients in Germany over time, (46) for which only speculative explanations can be put forward. Healthcare campaigns and lifestyle improvements might be partly responsible for this decrease.

Remarkably, our study suggests therapeutic hypothermia was underused in special patient groups, such as female and older STEMI patients with CPR, in spite of well-known less favorable outcomes after MI events in these groups (47–52). However, studies have reported an age-dependent effect of targeted temperature management on outcome after cardiac arrest, showing that therapeutic hypothermia was only associated with improved neurological outcomes in younger individuals; patients aged 65 years or older did not significantly benefit from this treatment (53). This observation may be responsible for the declining use of therapeutic hypothermia with patients after the fourth decade of life. Nevertheless, more than 10% of STEMI patients older than 75 years present with cardiogenic shock (51) and many randomized controlled trials regarding STEMI excluded elderly patients, and thus, the risks and benefits of revascularization therapies in this population were not elucidated (50–52). In terms of sex disparities, studies have shown that mortality is higher in women with STEMI than in men, which can be explained by their unfavorable risk profile and longer symptom-to-balloon time (48, 49). Remarkably, there was no significant difference in neurological outcomes or crude mortality outcomes between sexes in cardiac arrest survivors who received therapeutic hypothermia (54). In line with our study results, Morris et al. (55) reported that female resuscitated STEMI patients were less likely to be treated with therapeutic hypothermia (55). In this study, the explanation for the lower use of therapeutic hypothermia in female patients with cardiac arrest was that there was a higher percentage of women with do-not-resuscitate orders/family requests and non-shockable rhythms (55).

In summary, awareness of the optimization of therapy approaches using therapeutic hypothermia needs to grow when considering the treatment of STEMI patients after cardiac arrest. The increasing use of therapeutic hypothermia in STEMI patients after cardiac arrest in Germany from 2005 to 2019 might point to an increased awareness of its use in daily practice with these critically ill patients at the highest risk of death.

### Limitations

There were some limitations in this study that require consideration: (1) study results are based on ICD- and OPS-codes of hospitalized patients, which might be under reported or under coded; (2) although data for in-hospital stays are included, data for later follow up are not; and (3), as mentioned previously, the main limitation is that the exact timing and course of CPR (i.e., whether CPR was performed on admission or soon after hospitalization before cardiac catheterization, or during an in-hospital complication), and the timepoint of therapeutic hypothermia treatment, could not be exactly determined.

## Conclusion

Therapeutic hypothermia was associated with a survival benefit in hospitalized STEMI patients with CPR. However, only 12.3% of the STEMI patients with CPR were treated with therapeutic hypothermia. Therapeutic hypothermia was predominantly used in younger STEMI patients. Pneumonia and acute kidney injury occurred more often in STEMI patients treated with therapeutic hypothermia.

## Data availability statement

The datasets presented in this article are not readily available. Requests to access the datasets should be directed to karsten.keller@unimedizin-mainz.de.

## Ethics statement

Ethical review and approval was not required for the study on human participants in accordance with the local legislation and institutional requirements. Written informed consent from the participants' legal guardian/next of kin was not required to participate in this study in accordance with the national legislation and the institutional requirements.

## Author contributions

KK and LH were responsible for conceptualization, methodology, software and programming, validation, formal analysis, investigation, resources, data curation, visualization, project administration, and supervision. KK wrote the original draft. All authors contributed to the article and approved the submitted version.

## Conflict of interest

TG and TM are PIs at the DZHK (German Center for Cardiovascular Research), Partner Site Rhine-Main, Mainz, Germany. TG has received grant support (CARIMA study) and speaker's honoraria from Novartis, and speaker's honoraria from Boehringer Ingelheim, Daiichi-Sankyo, MSD, Pfizer – Bristol-Myers Squibb, and Astra Zeneca. LH received lecture honoraria from MSD. The remaining authors declare that the research was conducted in the absence of any commercial or financial relationships that could be construed as a potential conflict of interest.

## Publisher's note

All claims expressed in this article are solely those of the authors and do not necessarily represent those of their affiliated organizations, or those of the publisher, the editors and the reviewers. Any product that may be evaluated in this article, or claim that may be made by its manufacturer, is not guaranteed or endorsed by the publisher.
